# North-Eastern Hospital for Children

**Published:** 1906-05-19

**Authors:** 


### NORTH-EASTERN HOSPITAL FOR CHILDREN.

About 120 ladies and gentlemen attended the festival dinner of the North-Eastern Hospital for Children, held on Tuesday evening at the Grocers' Hall, which was kindly lent by the company for the purpose. In the unavoidable absence (owing to the death of the Duchess of Connaught's mother) of the Duke of Connaught, who was to have presided, Alderman Sir Marcus Samuel took the chair. The dinner resulted in a collection of £2,900 being realised.

After the toast of "The Houses of Parliament," proposed by Sir Joseph Dimsdale, Bart., and responded to by Earl Manners.

The Chairman proposed "The North-Eastern Hospital for Children." He first apologised for having to preside owing to the absence of the Duke of Connaught, to whom and to whose Duchess the hospital was enormously indebted for innumerable services rendered. For example, his Royal Highness had been connected with the hospital for many years, and in 1880 the Duchess, accompanied by the Duke, had opened the first specially constructed wards of the hospital which previously to that date had been carried on in a small embryo state. Wards containing 57 beds were later on opened by the Duchess of Connaught, whilst one of the wards bore her name. (Applause.) In 1891, on May 15, curiously enough, the Duke of Connaught presided over the dinner in aid of the hospital, at which dinner the amount collected was £2,100, a sum, he hoped, they would exceed that evening. In 1896 the Duchess of Connaught, who was accompanied by the Duke, opened a "bazaar of roses" *fête* for the hospital in the Queen's Hall, where £3,000 was collected, and in consequence of that the debt on the hospital at that time was almost liquidated.

### A Message from the Duke of Connaught.

He had received a message from the Duke of Connaught who wished him to tell those present that evening how extremely he regretted not being able to be with them. His Royal Highness was sure they would understand the reason for his absence, and he hoped the dinner would be a great success. (Applause.)

### The History of the Hospital.

The hospital had made its way up from very small beginnings. Started in 1867 by two Quaker ladies in Virginia Road, Bethnal Green, it in a very short time had to move to larger premises in Hackney Road. Various improvements continued to be made year after year until in 1872 it was removed to the present site. Opened with 57 beds in 1880, it continued its work for 20 years, but the requirements of hospital science made such enormous strides, the methods of hospital construction were so different nowadays, and the population of the district in which the hospital stood increased so swiftly that an extension became an absolute necessity. The district served by the hospital numbered over 500,000, and included Hackney with 225,000 inhabitants, Bethnal Green with 130,000, and Shoreditch with 118,000. In addition, children came to the hospital from Walthamstow, Tottenham, and Enfield. Although the necessity for an extension of the hospital was apparent in 1890, it was not until 1898 that the present Bishop of London (then the Bishop of Stepney) took the matter up, and in 1901 sufficient funds had been collected to justify the commencement of building operations. The building thus commenced was completed in 1903, and now (including the 57 beds in the old wards) contained 125 beds. The committee had so far expended £48,000 on this portion of the extension, and of this £8,200 was still owing, having been raised on mortgage at 4 per cent. This £8,000 included a sum of £5,000 which had been expended on a large site adjoining the hospital.

### Last Year's Work.

Last year the number of children admitted to the wards was 1,609, whilst 27,733 had received treatment in the out-patients' department, where they had made 75,637 attendances.

Colonel Lord William Cecil, the Chairman of the General Committee, responded.

Sir Henry C. Burdett proposed "The Medical, Surgical, and Nursing Staff." Before speaking to the toast directly, he said he would like to take the opportunity of pointing out that among the children's hospitals of London he knew no hospital which more deserved, and more warranted, support than the North-Eastern Hospital for Children. The position of the hospital alone gave it the premier claim upon the charity of the Metropolis. It was situated, as he knew from personal inspection, in a district where there were many children of the poor, and he claimed that amongst the hospitals of London, and especially among the children's hospitals, there was no better object to which they could give their money than the North-Eastern Hospital. In making that claim of course he endorsed the management of the institution. He endorsed it from knowledge, for he knew that in addition to the unanswerable claim of the needs of the district which that hospital served, its committee of management had shown notable enterprise which had won for it the approval of the most observant of those who took the deepest interest in the welfare of the hospitals in London. It had also the merit of being conducted with regard to sound finance. What, it might be said, of the quality of the work? Well, that must be judged, as they judged the character of a man or a woman, by the esteem in which it was held by those who knew it. (Hear, hear.) This hospital had the singular merit of occupying a unique position in that the leaders of religious thought in the district had spontaneously combined to form a committee to raise funds to enable the hospital buildings to be erected.

There was one other matter. By the addition of balconies they had added, as the medical profession had testified recently in the medical journals, immeasurably to the life and the vital powers of the children who came to the hospital. He regretted, however, to hear on the authority of the medical staff that although certain cases of chest diseases had improved by being treated for months in the open air on the balconies there were certain classes of cases where the heart was involved which it was impossible to treat at present because the local authorities of Bethnal Green were out of touch with the needs of the population. Otherwise wood pavement would be laid down in front of the hospital without a moment's avoidable delay. Did the members of the Bethnal Green Corporation realise that the absence of wood pavement was rendering the rapid cure of many children practically impossible? Let the committee see to it that this fact was brought to the knowledge of the Mayor of Bethnal Green at once, when surely the Corporation would act effectively and promptly.

### The Medical Staff.

They had, as those present knew, three physicians and two surgeons, two assistant physicians, two assistant surgeons, a physician for skin diseases, an ophthalmic surgeon, dental surgeon, and a pathologist and bacteriologist, and also a medical gentleman who superintended the electrical department. He mentioned these members of the staff because he wanted his hearers to understand that the managers of the hospital had covered the whole field of the care of children. (Applause.) He was sure those present that evening would wish to express their sense of indebtedness to Dr. Wills, the late senior physician, who had retired since the last dinner. He likewise had to cordially congratulate Dr. George Carpenter who had succeeded to the vacant physician's post.

### The Nursing Staff.

Of the nursing staff, he could say only good, and in the matron, Miss Bushby, they had a head who was known outside the hospital, and favourably known too.

Dr. James Taylor, the senior physician, responded, and other toasts followed.

